# A Thermochromic Superhydrophobic Surface

**DOI:** 10.1038/srep27984

**Published:** 2016-06-15

**Authors:** Pietro Cataldi, Ilker S. Bayer, Roberto Cingolani, Sergio Marras, Ryad Chellali, Athanassia Athanassiou

**Affiliations:** 1 Smart Materials, Istituto Italiano di Tecnologia, Via Morego 30, 16163 Genova, Italy; 2 Nanochemistry, Istituto Italiano di Tecnologia, Via Morego 30, 16163 Genova, Italy; 3 Pattern Analysis and Computer Vision (PAVIS), Istituto Italiano di Tecnologia, Via Morego 30, 16163 Genova, Italy

## Abstract

Highly enhanced solid-state thermochromism is observed in regioregular poly(3-hexylthiophene), P3HT, when deposited on a superhydrophobic polymer-SiO_2_ nanocomposite coating. The conformal P3HT coating on the nanocomposite surface does not alter or reduce superhydrophicity while maintaining its reversible enhanced thermochromism. The polymeric matrix of the superhydrophobic surface is comprised of a blend of poly(vinylidene fluoride-co-hexafluoropropylene) copolymer and an acrylic adhesive. Based on detailed *X*-ray diffraction measurements, this long-lasting, repeatable and hysteresis-free thermochromic effect is attributed to the enhancement of the Bragg peak associated with the *d*-spacing of interchain directional packing (100) which remains unaltered during several heating-cooling cycles. We propose that the superhydrophobic surface confines π–π interchain stacking in P3HT with uniform *d*-spacing into its nanostructured texture resulting in better packing and reduction in face-on orientation. The rapid response of the system to sudden temperature changes is also demonstrated by water droplet impact and bounce back on heated surfaces. This effect can be exploited for embedded thin film temperature sensors for metal coatings.

Most non-substituted conjugated polymers were not easily processed by solvents and thermoforming due to strong interchain interactions and chain stiffness. In order to solubilize them in common solvents, side chains were introduced such as alkyl side chains for polythiophenes or similar silicon containing side chains for soluble pentacene polymers. Hence, substituted conjugated polymers have become commonplace for various applications such as organic solar cells, field effect transistors, electrochromic windows and thin film sensors^1^,^2^,^3^,^4^,^5^. Attachment of relatively long and flexible side chains to conductive polymer backbones not only enables processing in solvents but also results in intriguing reversible color changes (chromism) under certain conditions, such as thermochromism, solvatochromism, piezochromism, and affinitychromism^6^,^7^,^8^,^9^. Among conductive conjugated polymers, poly(alkylthiophenes), have emerged as one of the most popular class of conducting polymers. In particular, a specific structure known as highly regio-regular 2– 5′ head-to-tail coupled poly(3-hexylthiophene) or P3HT, has advanced the technology of polymeric field-effect transistors to the extent that field-effect mobilities now reach 0.1 cm^2^V^−1^s^−1^ approximating that of α-Si^10^. P3HT in solution is highly sensitive to various parameters, such as temperature variations, light, and solvent type. Discovery of thermochromism in poly(3-alkylthiophenes) due to conformational changes dates back to the late 1980s^11^. Such alkyl-substituted polythiophenes are in fact semicrystalline polymers and melting of the crystalline sections due to heating is associated with drastic changes in the chain conformation. Melting causes disorder within the alkyl side chains disrupting planarity and long cWonjugation lengths^12^. Heeger and co-workers also showed that P3HT can maintain a liquid crystalline state after crystal melting^13^. Similarly, such order-to-disorder transformations are observed in solvent dispersions of P3HT due to temperature or solvent type accompanied by solution color changes (solvochromic effect)^14^,^15^,^16^. For instance, P3HT can form nanorods in poor solvents or when cooled in good solvents accompanied by color changes^15^. In good solvents P3HT can also be found in the form of a mixture of random coils and nanorods^17^. Dissolving P3HT in chloroform (a good solvent) gives clear light red-orange solutions that slowly change color on standing for long times. Adding a poor solvent to this solution causes a drastic color change of the polymer to blue-violet in transmission. Upon further addition of the good solvent, the polymer recovers its initial color.

Earlier studies showed that thin films of P3HT deposited on smooth glass or Si-wafer surfaces by spin coating from good solvents, are red-violet at room temperature, but when heated to temperature above 150 °C change color to light red-orange^18^. On cooling, they slowly recover their initial color. The process was reported to be sensitive to oxidation by ambient oxygen levels. During transition, two phases can be discerned, a disordered (red-orange) phase with a high density of defects, and an ordered (red-violet) phase with a low density of defects^18^. The transition from the disordered to the ordered phase is characterized by very long relaxation times.

It has been recently shown that bioinspired nanostructured surfaces drastically enhance thermochromism in inorganic thermochromic materials such as vanadium dioxide^19^. To the best of our knowledge, thermochromic changes in polythiophenes on such bioinspired surfaces or on soft polymeric nanostructured materials have not been studied so far. Many superhydrophobic surfaces have been developed following bioinspired strategies. Recent studies indicate that certain physicochemical phenomena can be enhanced on superhydrophobic surfaces such as electro-osmosis, diffuso-osmosis, electrolyte diffusion in fuel cells, surface enhanced Raman scattering and cell adhesion^19^,^20^,^21^,^22^,^23^. For instance, superhydrophobic surfaces, due to their sub-micrometer surface texture and hydrophobic chemistry, can confine random analyte liquid spreading into a much smaller area compared to hydrophilic platforms, tremendously enhancing Raman scattering signals^23^. By the same token, in this work, we investigate and report on the thermochromic properties of thin regioregular P3HT films deposited on hydrophobic polymer and superhydrophobic polymer/silica nanocomposite surfaces. We show that although solvent-cast regioregular P3HT films demonstrate thermochromism on a soft, smooth, and hydrophobic polymer surface, surprisingly, on a superhydrophobic polymer-silica nanocomposite surface, the thermochromism is remarkably enhanced with fast reversibility, excellent reproducibility and stability exceeding six months under ambient conditions.

## Experimental

The chosen hydrophobic polymer was poly(vinylidene fluoride-co-hexafluoropropylene), PVDF-HFP (average Mw ~400,000, average Mn ~130,000). It was purchased from Sigma-Aldrich. It is a crystalline copolymer with good thermal stability as well as good solvent resistance. PVDF-HFP copolymers are being extensively studied as new alternatives for traditional liquid electrolytes in application for lithium-ion batteries^24^,^25^,^26^,^27^,^28^,^29^. P3HT having greater than 90% head-to-tail regiospecific conformation used in this study was produced by Rieke^®^ Metals, Inc. Fumed hydrophobic silica nanoparticles (Aerosil R812S; 220 mg/m^2^, BET) treated with bis(trimethylsilyl)amine (HMDS) were obtained from Evonik Inc. Initially, PVDF-HFP pellets were dissolved in dimethylacetamide to form stock solutions containing 10% polymer by weight Spray solutions were prepared by diluting the stock solution with acetone to obtain solutions containing 3% polymer by weight. It was not possible to form pure PVDF-HFP coatings with firm substrate adhesion by spray. Spray-cast coatings of PVDF-HFP easily peeled off from aluminum substrates as free standing films after solvent evaporation and drying in an oven at 80 °C for one hour. In order to increase its metal substrate adhesion properties PVDF-HFP was blended in solution with an anaerobic cure acrylic adhesive (Loctite 270, Henkel) comprising polyethylene glycol dimethacrylate (65%), bisphenol A propylene oxide fumarate polymer (18%), poly (butyl methacrylate) (15%) and cumene hydroperoxide (2%). The approach followed was identical to earlier reports on blending fluorinated polymers with acrylic adhesives in solution to enhance final coating substrate adhesion as well as its abrasion resistance^30^,^31^. The best substrate adhesion performance was displayed by the polymer matrix comprising a blend of 50% PVDF-HFP and 50% acrylic adhesive by weight.

Superhydrophobic nanocomposites were made by spraying acetone solutions containing PVDF-HFP, acrylic adhesive and Aerosil R812S SiO_2_ nanoparticles (NPs) onto aluminum substrates. Various amounts of fumed silica nanoparticles were added to the polymer solutions containing both PVDF-HFP and the adhesive in acetone. Mild sonication (Clifton MU Series, Analogue, Unheated Ultrasonic Bath, 200 W, U.K.) was used for half an hour to ensure the nanoparticles were dispersed thoroughly in the polymer solutions. After spray coating, all the samples were left under ambient conditions overnight for solvent evaporation and subsequently thermally treated at 150 °C in an oven for 1 minute to ensure complete solvent evaporation. The hydrophobicity of the coatings was changed by varying the concentration of the nanoparticles with respect to the polymer matrix. The nanoparticle concentration was ranged from 1% to 30% by weight with respect to the polymer on dry basis. The best performing coating contained 20% by wt. nanoparticles. The coating demonstrated static water contact angles exceeding 160° with negligible contact angle hysteresis; at the same time having good substrate adhesion measured by tape peel tests (see Supporting Information, Figure S1).

Static water contact angles and droplet roll-off angles of the samples were measured by a video based optical contact angle measuring instrument DataPhysics, Germany. Ten microliter of deionized water was gently placed on the surfaces. Measurements were conducted on four different locations and averaged for each sample. In order to measure the RAs, the substrates were tilted until the droplets started to roll off the surfaces. The substrate angle at the onset of droplet roll-off was recorded. All RA values were averaged over three different measurements on each sample. The standard deviation was ±4° for the static contact angle measurements and ±1° for the droplet roll-off angle measurements. All measurements were performed in ambient conditions. Changes in the static water contact angle as a function of tape peel events were recorded by the same optical contact angle measurement system. For this purpose, a plastic 3 M tape with 820 N/m adhesion strength on steel was used as reported by the manufacturer. Tapes were cut in proper sizes and adhered on the surface of the coatings by hand applying downward pressure at the same time. After the tapes were peeled off, water droplets were deposited on those spots where the tape was applied and contact angles were recorded. Four measurements were taken after each peel event. Two different superhydrophobic surfaces were tested containing 20 wt% and 30 wt% SiO_2_ nanoparticles with respect to the polymer binder. None of the coatings could be completely removed at the end of 15 peel events indicating that the coating adhesion to aluminum was larger than 820 N/m. However, the coating with 30 wt% SiO_2_ nanoparticles demonstrated lower water contact angles close to the generally accepted superhydrophobicity threshold (150°).

The enhanced thermochromic effect was observed when a thin layer (~0.15 μm) of P3HT was sprayed over the cured superhydrophobic coatings. For this, a solution containing 0.1% by wt. P3HT in chloroform was prepared. This additional P3HT layer did not reduce superhydrophobicity of the coatings as it conformed to the underlying texture. After spraying P3HT the solvent was completely evaporated overnight under standard hood ventilation. Further details regarding the thermochromic effect measurements are given under the results and discussion section of this letter. Atomic force microscopy (AFM) images were obtained by a Park System AFM instrument (XE-100) in true noncontact mode. The images were acquired in air using an anti-vibration table (Table Stable TS-150) and an acoustic enclosure. Single-beam silicon cantilevers tips (PPP-NCHR-10) were used for the data acquisition with less than 10 nm nominal radius and 42 N/m elastic force constant for high sensitivity. The resonance frequency was defined around 280 kHz. The scan rate was between 0.2 and 1.0 Hz.

Melting and crystallization temperatures were measured by heating and cooling the sample from 20 to 250 °C at a rate of 10 °C min^−1^ using a differential scanning calorimeter (TA Instruments, 2920 Modulated DSC).

The crystal structure was studied by X-ray diffraction (XRD) using a Rigaku SmartLab X-Ray diffractometer, equipped with a 9 kW Cu Kα (λ = 1.542 Å) rotating anode, operating at 40 kV and 150 mA. A Göbel mirror was used to convert the divergent X-ray beam into a parallel beam and to suppress the Cu Kβ radiation (λ = 1.392 Å). The diffraction patterns were collected at room and elevated temperatures, over an angular range of 4° to 35°, with a step size of 0.05° and scan speed of 1.2°/min. Grazing Incidence X-ray diffraction (GI-XRD) measurements were conducted through University of Illinois beam time at the 12-ID-C beamline (energy = 11.26 keV, pixel size = 79.6 μm, wavelength = 1.101 Å, *2*θ = 0 − 20° at Argonne National Laboratory, USA.

The thermochromic response of the P3HT films on the superhydrophobic nanocomposites was measured using diffuse reflection spectroscopy with an integrating sphere attachment to UV-Vis-NIR spectrophotometer (Varian Cary 6000i). The measurements were performed by placing the samples in front of the incident light window, and concentrating the light reflected from the sample on the detector using a sphere having a barium sulfate-coated interior. The obtained value becomes the reflectance (relative reflectance) with respect to the reflectance of the reference standard white board, which is taken to be 100%. A flat resistive heater was attached on the back side of the coated aluminum substrates in order to collect reflection spectra as a function of temperature (in steps of 10–15 ° C).

## Results and Discussions

**Figure 1:**
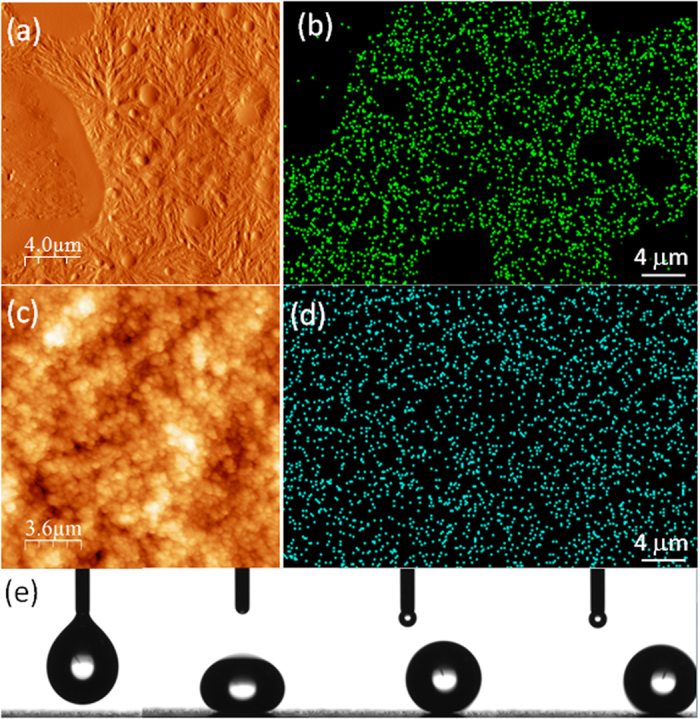
(**a**) AFM surface topography of the hydrophobic PVDF-HFP/adhesive coating. Randomly dispersed mostly spherical and smooth regions are attributed to the adhesive. (**b**) Fluorine EDX signal form the same surface (not exactly the same location). Dark regions correspond to cured adhesive domains. (**c**) AFM surface topography of the superhydrophobic polymer-silica nanocomposite. (**d**) Fluorine EDX signals form the same surface (not exactly the same location) showing much more uniform signal distribution. (**e**) An image sequence showing a water droplet (~10 μL) being dispensed on the superhydrophobic surface and its subsequent oscillation and rolling off the surface.

Figure 1 shows atomic force microscope (AFM) topography and energy dispersive X-ray mapping (EDS) of the fluorine atom distribution pertaining to PVDF-HFP/adhesive polymer surface (hydrophobic surface) and the superhydrophobic nanocomposite (with 20% wt. hydrophobic SiO_2_ NPs) prior to P3HT deposition.

**Figure 2:**
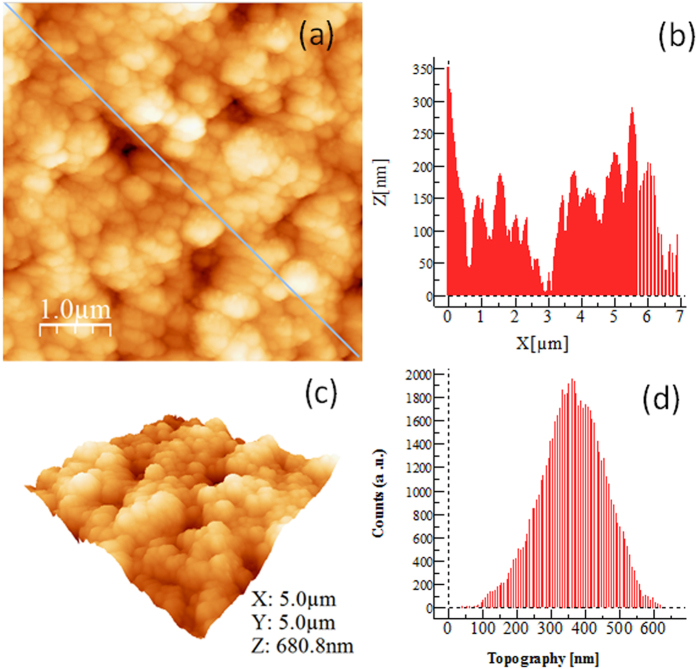
(**a**) Atomic force microscope (AFM) topography of the superhydrophobic polymer-silica nanocomposite coated with a P3HT which conforms to the texture. The diagonal line is used to obtain a random roughness profile on the surface shown in (**b**). (**b**) Roughness profile corresponding to the diagonal line in (**a**). The deepest valley is designated as zero. (**c**) 3D topological features of a 5 × 5 μm thermochromic superhydrophobic surface. (**d**) Normal (Gaussian) roughness distribution obtained from such as surface with an average roughness of 350 nm.

In general, the adhesive forms random micron-scale phase separated domains within PVDF-HFP as shown in Fig. 1a,b. The superhydrophobic film appears to have a dual scale hierarchical topology as can be seen in Fig. 1c. The fluorine atom distribution of this surface (Fig. 1d) is much more uniform compared to the unfilled polymer matrix (Fig. 1b). The degree of superhydrophobicity of this surface is demonstrated by a sequence of images collected during the deposition of a 10 μL water droplet in Fig. 1e. It is practically very difficult to maintain a water droplet on such surface as a slight tilt causes the droplet’s roll-off. Note that the superhydrophobicity is maintained when the surface was coated with P3HT. Further surface topography analysis of the superhydrophobic surfaces coated with P3HT was also made by AFM. Results indicate that surface roughness of the polymer-silica nanocomposite is preserved as seen in Fig. 2a. Typical roughness profile on such a surface is shown in Fig. 2b. The roughness profile is obtained from the diagonal line shown in Fig. 2a where the deepest valley is set to zero in the plot (Fig. 2b). Based on the measurements from different samples of identical nature, we found that the maximum surface roughness value does not exceed 450 nm. A 3D AFM topology is exemplified in Fig. 2c. The normal roughness distribution of the thermochromic superhydrophobic surface shown in Fig. 2d was found to be narrow and remained below 1 μm with an average roughness of ~350 nm. Such a roughness profile is sufficient to create the self-cleaning “lotus effect” when combined with a uniformly formed hydrophobic surface chemistry as shown in Fig. 1d in the form of EDS fluorine signals.

**Figure 3:**
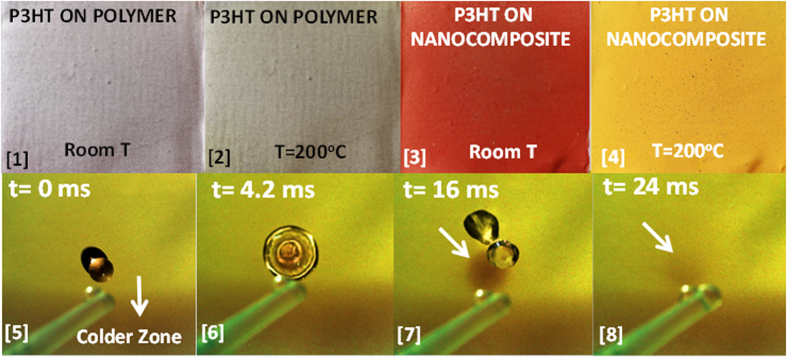
Top row: [**1**] P3HT layer on hydrophobic PVDF-HFP/adhesive coating on aluminum at room temperature, [**2**] P3HT layer on hydrophobic PVDF-HFP/adhesive coating on aluminum at 200 °C, [**3**] P3HT layer on superhydrophobic polymer-silica nanocomposite on aluminum at room temperature, and [**4**] P3HT layer on superhydrophobic polymer-silica nanocomposite on aluminum at 200 °C. Panels [**5–8**] are sequence of a water droplet impacting on a hot surface ~200 °C. The droplet was released from plastic pipette (visible in the images). The lower section of the surface is cooler than the center as that edge of the coated aluminum was not in contact with the hot plate surface in order to enable handling during experiments. When the droplet makes impact, due to cooling, the color of the zone under the droplet immediately changes to purple-red. Video of this sequence is available in electronic supplementary information.

Figure 3 shows the thermochromic behavior of a P3HT layer deposited on both the hydrophobic surface polymer surface (Fig. 3[1]), and on the superhydrophobic nanocomposite (Fig. 3[3]), at temperatures between room temperature and 200 °C (obtained on a hot plate under ambient conditions). Thermal conductivity of aluminum is very high (215 Wm^−1^K^−1^ at 25 °C) enabling rapid heat transfer from the hot plate to the coating layers. As can be seen in Fig. 3, for the P3HT applied on the hydrophobic polymer film, the color change perception is not significant once the film is heated to 200 °C. P3HT on the superhydrophobic nanocomposite surface, however, displays a distinct purple-red color at room temperature that changes to light orange-yellow color at 200 °C. The degree of superhydrophobicity of the P3HT coated nanocomposite surface and its response to sudden temperature changes are demonstrated in Fig. 3 ([5]–[8]). A sequence of high speed camera images is shown, in which a water droplet impacts on the surface maintained at 200 °C. As the room temperature droplet impacts and spreads, the color of the contact region immediately turns purple-red indicating the rapid response of the system to the changes in temperature. Once the droplet bounces away from the surface, the color of the region underneath the droplet impact turns back to light orange-yellow. Upon impact, water droplet causes a decrease in the temperature of the surface and this is reflected as a local color change due to re-crystallization and macromolecular rearrangement due to cooling. Note that during all this process the surface still maintains its superhydrophobicity. In fact, according to Ewbank *et al*.^32^, in all the solid phases of P3HT such as crystalline, meso, or kinetically trapped phase, the hydrophobic alkyl side chains always orient perpendicular to the plane of the substrate. Only the case of “face-on” morphology forces the waxy hydrophobic side chains to orient parallel to the substrate. It is therefore highly probable that during crystallization and degradation of crystal domains on the superhydrophobic surfaces the “face-on” orientation does not occur but rather the morphological changes can pertain to transformation from crystalline to meso or to kinetically trapped phase.

**Figure 4:**
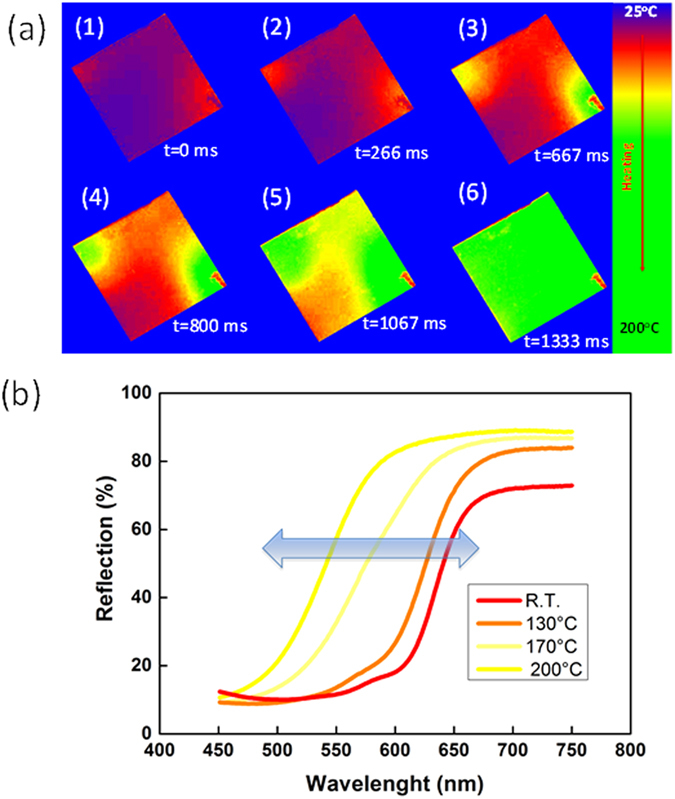
(**a**) High speed camera image sequences (600 fps) showing heating and resulting color change of the thermochromic superhydrophobic surface. The blue background corresponds to room temperature and green to 200 °C as a result of image processing for better perception. The red smudge on the lower right corner is the uncoated region for sample handling. (**b**) Temperature dependent shift in the surface reflection measured by diffuse reflection spectroscopy between 450–750 nm.

Figure 4a demonstrates the evolution of the color change of the P3HT coated superhydrophobic nanocomposite containing 20% wt. SiO_2_ NPs. The event was recorded by a high speed camera at 600 fps when the sample was suddenly placed on a hot plate maintained at 200 °C. The recorded frames were processed by a color tracking algorithm. For enhanced perception, the algorithm assigns blue to room temperature and green to 200 °C. The heating of the plate starts from two opposite corners and propagates to cover the whole plate within 1.3 seconds. The initiation of the heating from the corners can be attributed to the plate’s curvature. The observed thermochromic response of the P3HT films on the superhydrophobic nanocomposites was measured using diffuse reflection spectroscopy with an integrating sphere attachment to UV-Vis-NIR spectrophotometer (Varian Cary 6000i). The change in the reflectance spectra between 450 nm to 750 nm is displayed in Fig. 4b. As the temperature increases the spectra show a blue shift and at the same time the reflectance intensity increases at all wavelengths. The shifts in the spectra due to heating were followed by recording the changes in the wavelength corresponding to 45% reflection that is approximately half of the reflection range. The shifts were denoted by Δλ_R1/2_ for every temperature step. Alternatively, the backward shift of the thermochromic coatings was also recorded during cooling back to room temperature to gauge the degree of repeatability as well as the presence of any hysteresis.

**Figure 5:**
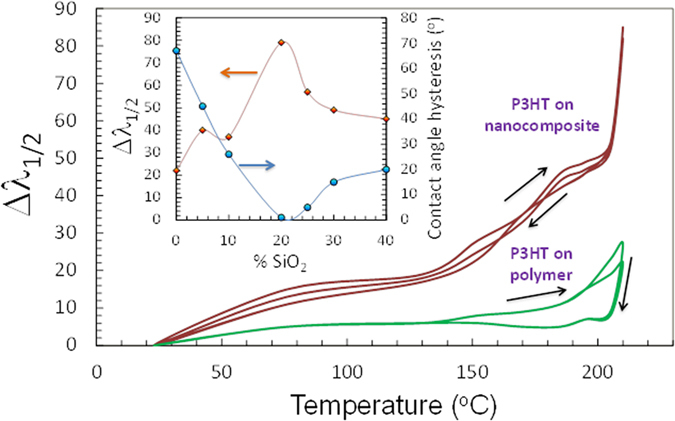
Changes in the spectra as a function of temperature traced by recording the changes in the wavelength corresponding to 45% reflection. The shifts were denoted by Δλ_R1/2_ for every temperature step. The hysteresis is also shown by cooling down the heated surfaces for P3HT on the superhydrophobic surface and on the polymer coating alone. The inset shows the value of Δλ_R1/2_ and water contact angle hysteresis as a function of SiO_2_ nanoparticle concentration within the polymer matrix.

In the case of P3HT on the hydrophobic coating, the shift Δλ_R1/2_ is less than 10 nm until 175 °C and becomes 30 nm at 200 °C (see Fig. 4). Upon cooling, however, a Δλ_R1/2_ of 10 nm is suddenly obtained and then no more change is detected until room temperature is reached. Upon heating again, a similar trend in Δλ_R1/2_ is observed but at the maximum temperature a lower Δλ_R1/2_ value is reached compared to the first heating cycle. After the fifth cycle, Δλ_R1/2_ practically remains constant indicating termination of thermochromism. On the contrary, when P3HT was deposited on the superhydrophobic surface, the Δλ_R1/2_ changes were found to be highly repeatable with slight hysteresis for both heating and cooling cycles. During heating, the wavelength shift Δλ_R1/2_ is 20 nm at 95 °C, and remains somewhat steady until 130 °C. Above this temperature an almost linear increase of the shift occurs until 200 °C and at 200 °C suddenly Δλ_R1/2_ jumps to 85 nm (maximum shift). During cooling a similar trend was observed and the heating-cooling cycles were stopped after twenty loops with no visible deterioration in thermochromism. Effect of hydrophobic SiO_2_ NPs concentration on the observed shifts in the reflective spectra was also studied. The maximum shift in Δλ_R1/2_ was found to correlate with the amount of SiO_2_ NPs as shown in the inset of Fig. 5. The superhydrophobic surface with 20% wt. SiO_2_ NPs concentration demonstrates minimum contact angle hysteresis (see also Fig. 1e) as well as a maximum shift in Δλ_R1/2_. This means that thermochromism of the P3HT on these non-wetting surfaces is optimum in terms of perceptional color changes. Although surfaces with more than 20 wt% by wt. nanoparticle content were still superhydrophobic and with reasonable perceptional color changes, their tape-peel resistance (durability) decreased (see electronic Supplementary Information Figure S1).

**Figure 6:**
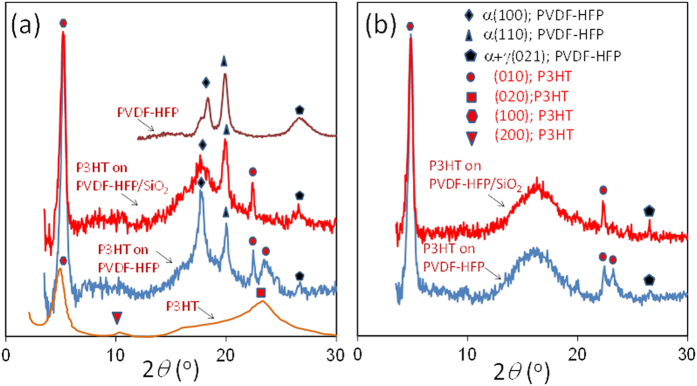
(**a**) Room temperature XRD results of P3HT and PVDF-HFP/adhesive polymer spray coated on aluminum and from P3HT coated on PVDF-HFP/adhesive polymer and on the superhydrophobic nanocomposite. (**b**) XRD results of P3HT coated on PVDF-HFP/adhesive polymer and the superhydrophobic nanocomposite at 200 °C.

Both PVDF-HFP and P3HT are semi-crystalline polymers and their crystallinity is well studied^32^,^33^,^34^,^35^,^36^. According to the DSC measurements, P3HT used in this study melts at around 213 °C upon heating and it crystalizes at about 173 °C. Hence, the highest temperature used in this study (200 °C) is very close but does not exceed the melting temperature of P3HT. The crystallinity of PVDF-HFP copolymer is lower than PVDF but it retains sufficient mechanical stability to allow it to act, for example, as a separator between the electrodes of a lithium-ion battery, while the amorphous phase can contain the liquid electrolyte^37^,^38^. The XRD spectrum of the hydrophobic coatings is shown in Fig. 6a. The separate XRD measurements of the pure adhesive yielded no crystallinity. Within the coating, PVDF-HFP is mostly in α crystalline form with non-polar trans–gauche–trans–gauche′ (TGTG′) conformation. However, the band at *2θ* = 26.7° indicates some presence of the γ phase which has an intermediate polar TTTGTTTG′ conformation, occurring when the polymer is moderately stressed^38^. The polymer here was not mechanically stressed; however, the presence of random but mostly spherical adhesive domains shown in Fig. 1 can induce various levels of internal stress to PVDF-HFP resulting in the partial appearance of the γ phase^39^. When the PVDF-HFP/adhesive polymer matrix is kept at 200 °C, the well-defined α (100) at 2*θ* = 18.4° and α (110) peaks at 2*θ* = 20° disappear indicating an amorphous state while some of the γ phase with α + γ(021) still retains its form *2θ* = 26.6° (results not shown)^28^.

As a result of various carefully conducted studies, there is now a consensus on a model for the ideal crystallinity of poly(alkylthiophenes) in which stacks of poly-conjugated main chains organize in layers, with the side chains extending in the regions between the stacked main chains^33^,^34^,^35^,^36^,^37^. When P3HT is spray-coated from a chloroform solution on aluminum with subsequent solvent evaporation and short thermal curing, it gives rise to an X-ray diffraction pattern as shown in Fig. 6a. The band at 2*θ* *=* 6.9–7.0° is normally indexed as (100) in the literature corresponding to the characteristic periodicity between adjacent layers of chains with non-interdigitating side chains. The present polymorph shows a broad diffraction pattern in the region 2*θ* = 23°–28° which can be indexed as (020). P3HT layers spray deposited on the hydrophobic coating and on the superhydrophobic nanocomposites, however, displayed significantly different changes in the X-ray diffraction patterns at room temperature. Particularly, at room temperature on hydrophobic coatings, the (100) diffraction signal was enhanced considerably and the broad (020) Bragg signal transformed into two bands (a narrow one at 2*θ* = 22.4° and broader one at 2*θ* = 23.5°) that can be indexed by (010) as seen in Fig. 6a. These out-of-plane (010) reflections due to π–π interchain stacking correspond to inter-chain distances of *d*_[010]_ = 3.81 Å and *d*_[010]_ = 3.96 Å, respectively. It is unusual to observe the splitting of the broad (020) peak which is characteristic of the spray cast films on aluminum. However, this sort of changes in crystallinity was observed before in polyalkylthiopehenes. Prosa *et al*. attributes this splitting of the board halo of (020) to “side chain disorder”^13^. Similar crystallinity disorder observations were also reported by Wu *et al*. later on^11^. This unusual structural behavior can be ascribed to layers of interdigitated, tilted alkyl chains having intra-stack perpendicular chain-to-chain spacing and a dense side chain nearest neighbor spacing^37^,^38^,^39^,^40^. As such, depending on the intra-stack chain repeat length and orientation, the broad (020) halo can be split into various reflections. Herein we denote the split reflections on the hydrophobic polymer with (010) as these could have mixed indices and in reality indexing them is quite hard as indicated by Yuan *et al*.^37^

The enhancement in intensity of the (100) diffraction signal on the hydrophobic coatings indicates a higher degree of crystallinity in P3HT as well as the absence of smaller or scattered domains in which the polymer orientation is random or disordered^41^,^42^,^43^,^44^. Foong *et al*. reported that nano-confinement imposes changes in the crystallinity (reduction) and the orientation of the P3HT polymer^45^. For heterojunction solar cells, this is considered non-ideal for transport of charges from the exciton dissociation interface to the electrodes^44^,^45^. They observed that P3HT molecules within TiO_2_ nanotube arrays were less crystalline than unconfined P3HT molecules with reduction in the intensity of the *d*_[100]_ peak^42^. They also indicated that hydrophilicity of the underlying substrate imposes changes in P3HT configuration. On hydrophilic surfaces, P3HT would assemble over its backbone rather than the hydrophobic alkyl side-chains^45^,^46^.

The inter-chain distance *d*_[100]_ of the P3HT packing on the hydrophobic surface is 16.6 Å, which corresponds to the typical P3HT interchain packing distance for a lamella packing pattern reported in the literature^45^,^46^,^47^,^48^,^49^,^50^. In the case of the P3HT layer on aluminum the *d*_[100]_ is 18.4 Å, higher than the generally accepted lamella packing value of 16.6 Å. The reduced *d*-spacing of the first Bragg peak of P3HT on the hydrophobic surface indicates that P3HT is better packed along the interchain direction. When the P3HT is applied on the superhydrophobic nanocomposite containing 20% wt. SiO_2_ NPs, at room temperature, the (100) Bragg peak is preserved well with corresponding *d*_[100]_ = 16.6 Å. This means that the superhydrophobic nanostructured texture does not disrupt the interchain packing distance. As mentioned earlier, the broad halo of P3HT indexed by (020) on the hydrophobic polymer splits into two peaks, one narrow and one slightly broader resembling the original halo on the hydrophobic polymer. On the superhydrophobic nanocomposite, this somewhat broader peak also vanishes. This might correspond to an “edge-on” type orientation when P3HT is deposited on the superhydrophobic surface textured mainly by the SiO_2_ nanoparticles. According to the review by Brinkmann^50^, the edge-on orientation reveals clear (010) reflection whereas in the case of mostly “face-on” type structure, the (010) reflection vanishes. Similarly, Yuan *et al*.^37^ indicated that edge-on or face-on orientation along with the side chain intra-alignments can produce sharp or diffused and split (010) reflections depending on the hybrid morphology, respectively. As seen, the broad peak at 2*θ* = 23.5° disappears on the superhydrophobic surfaces, whereas the narrow peak at *2θ* = 22.4° is strong. Hence, the π–π interchain stacking conforms to a single distance at *d*_[010]_ = 3.96 Å, indicating that on the superhydrophobic surface at room temperature, P3HT chains are probably packed more uniformly along the interchain direction^39^,^40^,^41^. When the P3HT layer on pure aluminum surface was heated to 200 °C, the (100) and (020) Bragg peaks significantly lost their intensity (not shown for brevity) but after cooling down to room temperature both crystalline indices reappeared. This is a well-known behavior related to the loss of π-stacking order due to melting of the crystals^45^,^46^,^47^,^48^,^49^,^50^. Upon heating P3HT coated hydrophobic polymer and superhydrophobic nanocomposite surfaces to 200 °C (Fig. 6b), the Bragg peaks of PVDF-HFP associated with α(100) and α(110) are replaced by a much broader signal centered around *2θ* = 16°, as a result the polymer loses its trans–gauche–trans–gauche′ (TGTG′) conformation. P3HT on the polymer, on the other hand, maintains the Bragg peaks at (100) as well as the two peaks associated with the (010) plane observed at room temperature. The splitting of the (020) plane into two (010) planes at 200 °C is better defined, particularly, the original signal at *2θ* = 23.5° is narrower and shows a slight shift to 23.1°, indicating formation of more uniform π–π interchain stacking distances. On the hot superhydrophobic nanocomposite surface, the first Bragg peak of the P3HT maintains a strong d-spacing, *d*_[100]_, still at 16.6 Å. The second Bragg (010) diffraction peak at *2θ* = 23.1° is absent, like in the case of the room temperature analysis, and the (010) peak at *2θ* = 22.4° is preserved. At the same time, the small Bragg diffraction signal at *2θ* = 26.6° pertaining to the γ phase of PVDF-HFP gets stronger. Summarizing, on the hydrophobic polymer surface or on the superhydrophobic nanocomposite surface, the inter-chain distance *d*_[100]_ of the P3HT packing is strong and reduced compared to P3HT on aluminum and does not shift between heating and cooling, remaining very stable. On the other hand, the broad out-of-plane (020) reflection due to π–π interchain stacking of P3HT on aluminum transforms into two narrow out-of-plane π–π interchain stacking that can be indexed by (010) on PVDF-HFP polymer. The suppression of one of these reflection at *2θ* = 23.5° indicates reduction of polymorphism in regioregular P3HT and the fact that it also occurs at 200 °C indicates highly stable polymer chain stacking of P3HT on the nanocomposites.

**Figure 7:**
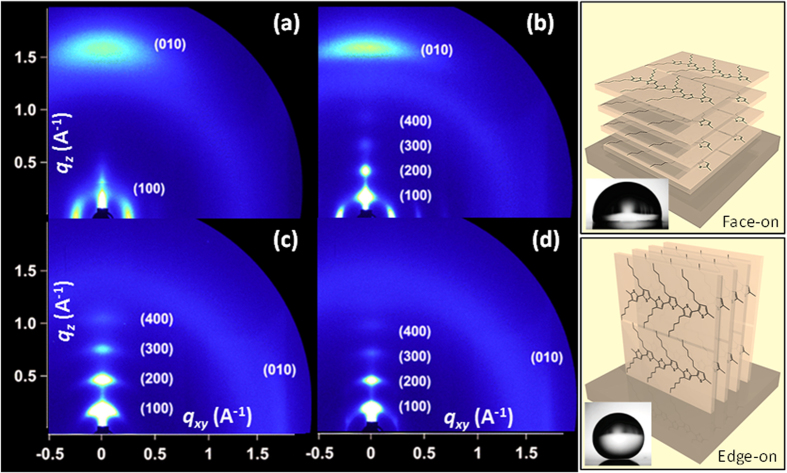
Room temperature grazing-incidence X-Ray diffraction (GIXRD) measurements and schematics representing face-on and edge-on orientation of P3HT. 2D GIXRD pattern of (**a**) P3HT deposited on bare aluminum surface, (**b**) P3HT deposited on PVDF-HFP/adhesive coating, (c) P3HT deposited on the superhydrophobic coating and (**c**) room temperature 2D GIXRD pattern on P3HT deposited on the superhydrophobic surface after cooling down from 10 seconds of 200 °C exposure. The panel next to (**b**) represents a face-on orientation of P3HT which is generally associated with hydrophobic surface (see inset) and the panel next to (**d**) represents the edge-on orientation which is associated with superhydrophobicity due to the orientation of the hydrophobic alkyl chains (see inset).

In Fig. 7, room temperature grazing-incidence X-Ray diffraction (GIXRD) patterns are shown. The 2D GIXRD images enable probing of the molecular-scale packing of the polythiophene on a particular surface, and are particularly sensitive to the efficient organization of P3HT in two orthogonal directions: the π–π stacking of the aromatic thiophene rings, and the lamellar stacking resulting from the disordered alkyl side-chains. Note that the third orthogonal direction (along the polymer backbone) does not always yield well-defined GIXRD patterns. Moreover, existence of face-on crystals on the hydrophobic or superhydrophobic films are not detected by 1D XRD measurements since their scattering vector is parallel to in-plane direction^51^. The intensity along the scattering rings can also be used to quantify the orientation distribution of the material, and thus the fractions of materials oriented in different directions. Figure 7a shows the room temperature crystalline state of spray coated P3HT film on a bare aluminum surface corresponding to the panel [1] of Fig. 3. X-ray reflections due to the (h00) crystal planes are not very clear except (100) plane. The (010) plane along the *q*_z_ axis is rather broad and is located close to the *q*_z_ axis indicating a face-on orientation. When P3HT is deposited on the polymer/adhesive coating (panel [2] in Fig. 3) however, the (h00) crystal planes are better defined although higher order ones such as (300) and (400) are very diffuse and of low intensity as seen in Fig. 7b. The (010) crystal plane appears to be located at the *q*_z_ axis but with less diffuse more intense nature indicating that the hydrophobic polymer surfaces induces a better and more ordered face-on orientation of P3HT confirming the experimental observation of Kline *et al*.^51^ (see panel next to Fig. 7b). On the superhydrophobic surface, the higher order (h00) crystal planes which correspond to the intermolecular backbone layer and π–π stacking order are somewhat defined close to the *q*_z_ axis, however an abrupt shift of the (010) plane to the *q*_xy_ axis (although with lesser intensity, Fig. 7c) indicates some P3HT molecules assumed an edge-on chain conformation with side chains oriented in a standing-up configuration on the superhydrophobic surface. Upon heating the surface to force a color change and later cooling down to room temperature (Fig. 7d), the resultant crystalline order practically appears similar to the state shown in Fig. 7c, with less diffused higher order (h00) crystal planes. The (010) maintains its position near the *q*_xy_ axis indicating the presence of edge-on orientation of the P3HT (see panel next to Fig. 7d). Although a more in-depth crystallographic analysis of these surfaces is beyond the scope of this work both 1D and 2D XRD measurements show that on the superhydrophobic surface at least some portion of the P3HT polymer assume an edge on orientation evidenced by the disappearance of the (010) plane near the *q*_z_ axis. However, P3HT coating also conforms to the submicron roughness associated with the superhydrophobic surface, rendering data collection and interpretation more challenging compared to for instance, a spin coated poly (3-alkylthiophene) crystals on a smooth surface.

As such, long-lasting thermochromic superhydrophobic surfaces which can function reversibly many times can find a wide range of applications particularly for metallic surfaces. Some potential and immediate applications can be in the form of smart non-wetting coatings for heat exchanger surfaces, waterproof temperature indicators or sensors for reactors, or outdoor storage enclosures to name a few. Future work can focus on various aspects related to use of other thermochromic organic molecules or polymers and also on fundamental aspects related to the assembly of the thermochromic polymers on superhydrophobic composite surfaces with submicron roughness scales.

## Conclusions

In conclusion, regioregular P3HT displays enhanced, long-lasting, and repeatable thermochromic effect when deposited on a superhydrophobic polymer nanocomposite comprising PVDF-HFP and hydrophobic SiO_2_ NPs. The first P3HT Bragg peak associated with the *d*-spacing of interchain directional packing is highly enhanced both on the hydrophobic and the superhydrophobic nanocomposites, indicating better crystalline state compared to untreated aluminum surfaces. This does not change when the system is heated to 200 °C. Moreover, the nanocomposite surface imposes interconversions within the out-of-plane π–π interchain stacking of P3HT resulting in better packing and reduction in polymorphism. We propose that the perceptional enhancement in P3HT thermochromism and most importantly its stability and non-hysteretic repeatability is due to the existence of π–π interchain stacking with uniform *d*-spacing confined into a nanostructured surface texture. It has been reported that P3HT does not lose its functionality when it assembles into superhydrophobic structures on textured surfaces or in composites as it is also the case in the present study. Previously superhydrophobic P3HT surfaces demonstrated effective electrical properties as well as photo-response while preserving self-cleaning properties^52^,^53^. In this work, we also demonstrated that it can maintain its solid-state thermo-responsive characteristics on superhydrophobic surfaces. This effect could be useful for designing thin film temperature detectors particularly for metal surfaces simply by spray coating. Future work will be conducted on better understanding of confinement of such thermochromic molecules bioinspired surface textures.

## Additional Information

**How to cite this article**: Cataldi, P. *et al*. A Thermochromic Superhydrophobic Surface. *Sci. Rep*. **6**, 27984; doi: 10.1038/srep27984 (2016).

## Supplementary Material

Supplementary Information

Supplementary Video S1
