# Supplementary material for: A Thermochromic Superhydrophobic Surface
Source: Sci Rep. 2016 Jun 15;6:27984. doi: 10.1038/srep27984 (PMC4908389; doi:10.1038/srep27984)
Supplement: Supplementary Information [file srep27984-s1.pdf]

Supplementary Information

A Thermochromic Superhydrophobic Surface

Pietro Cataldi,[a] *Ilker S. Bayer,*[a]* *Roberto Cingolani,*[b]* *Sergio Marras,*[c] *Ryad Chellali*[d], *Athanassia Athanassiou*[a]

[a]Smart Materials, Istituto Italiano di Tecnologia, Via Morego 30, 16163 Genova, Italy

[b]Central Laboratories, Istituto Italiano di Tecnologia, Via Morego 30, 16163 Genova, Italy

[c]Nanochemistry, Istituto Italiano di Tecnologia, Via Morego 30, 16163 Genova, Italy

[d]Pattern Analysis and Computer Vision (PAVIS), Istituto Italiano di Tecnologia, Via Morego 30, 16163 Genova, Italy

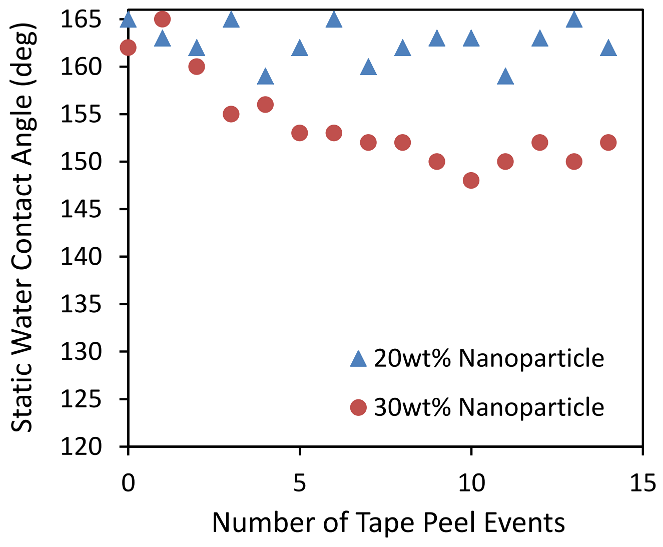

Figure S1. Changes in the static water contact angle as a function of tape peel events by using a 3M tape with 820 N/m adhesion strength on steel. Two different superhydrophobic surfaces were tested containing 20wt% and 30wt% SiO_2 nanoparticles. None of the coatings could be completely removed at the end of 15 peel events indicating that the coating adhesion to aluminum is larger than 820N/m. However, the coating with 30wt% SiO_2 nanoparticles demonstrated lower water contact angles close to the generally accepted superhydrophobicity threshold (150°).

Video File: Water droplet impact on a thermochromic superhydrophobic surface. Room temperature drop bounces on the heated superhydrophobic surface. The zone where the droplet impacts changes color due to temperature difference between the droplet and the surface.
